# Community Perspectives on Communicating About Precision Medicine in an Alaska Native Tribal Health Care System

**DOI:** 10.3389/fcomm.2020.00070

**Published:** 2020-09-25

**Authors:** R. Brian Woodbury, Julie A. Beans, Kyle A. Wark, Paul Spicer, Vanessa Y. Hiratsuka

**Affiliations:** 1 Southcentral Foundation, Anchorage, AK, United States,; 2 University of Oklahoma, Norman, OK, United States

**Keywords:** health communication, precision medicine, community-based research, Alaska Native, American Indian

## Abstract

**Background:**

Precision medicine seeks to better tailor medical care to the needs of individual patients, but there are challenges involved in communicating to patients, health care providers, and health system leaders about this novel and complex approach to research and clinical care. These challenges may be exacerbated for Alaska Native and American Indian (ANAI) people, whose experiences of unethical research practices have left some ANAI communities hesitant to engage in research that involves extensive data-sharing and diminished control over the terms of data management and who may have distinct, culturally-informed communication needs and preferences. There is need for communication research to support Tribal health organizations and ANAI people as they consider implementation of and participation in precision medicine. To address that need, this study characterizes the informational needs and communication preferences of patients, providers, and leaders at an Alaska Native Tribal health organization.

**Methods:**

We conducted 46 individual, semi-structured interviews to explore perspectives on precision medicine and related communication needs among patients, providers, and leaders of a Tribal health organization. Analysis involved team-based coding to identify a priori and emergent themes, followed by identification and recoding of content relevant to precision medicine informational needs and communication preferences.

**Results:**

Patients, providers, and leaders were described as both sources and recipients of information about precision medicine. Information deemed essential for making decisions about whether to participate in or implement a precision medicine program included information about the clinical and research applications of precision medicine, benefits and risks, health system costs and impacts, and data management practices. Preferred communication channels included digital and non-digital informational materials, as well as in-person learning opportunities for individuals and groups. Participants also describe contextual factors and barriers that influenced the acceptability and effectiveness of approaches to health communication.

**Conclusion:**

Results can inform approaches to communicating information about precision medicine to stakeholders within Tribal and other health care systems considering implementation of precision medicine in clinical or research contexts.

## INTRODUCTION

### Precision Medicine and Alaska Native and American Indian People

Precision medicine is an “emerging approach for disease treatment and prevention that takes into account individual variability in genes, environment, and lifestyle for each person” (NIH, 2019a). Proponents of precision medicine claim that precision medicine will improve health care quality and patient outcomes and point to recent successes in the clinical application of pharmacogenetics and gene therapies as evidence that these promises will be fulfilled (NIH, 2019b). At the same time, researchers and communities alike have raised concerns about the potential for precision medicine to undermine individual autonomy over personal health information, to expose individuals and communities to privacy risks and associated harms, and to exacerbate existing health disparities (Beskow et al., 2018; Jones et al., 2018; Stiles and Appelbaum, 2019). This tension between optimism and concern about the impacts of precision medicine is exacerbated for Alaska Native and American Indian (ANAI) people, for whom this emerging approach to clinical care and research simultaneously holds promise for addressing entrenched health disparities and is overshadowed by prior experiences of unethical research practices.

ANAI people are underrepresented in the clinical trials that lead to development of new treatments, pharmaceutics, and diagnostic tests (Murthy et al., 2004; LaVallie et al., 2008; Sprague et al., 2013). As a result, clinical best practices and advances in medical care and testing based on such research may fail to benefit or even pose health risks for ANAI people, exacerbating preexisting health disparities (Shaw et al., 2013; Obermeyer et al., 2019; Perera, 2019). Given the failure of standardized medicine to adequately account for their unique health needs and preferences, precision medicine-which promises an individualized approach to clinical care-may hold particular value for Tribal communities. For example, pharmacogenetic research with ANAI people has identified gene variants that impact nicotine metabolism (Claw et al., 2019), Type 2 diabetes diagnosis (Manousaki et al., 2016), and response to warfarin and other medications (Henderson et al., 2018, 2019). These findings have implications for treatment and disease diagnosis and provide case examples of how participation in precision medicine research could plausibly translate into positive changes in clinical care and health outcomes.

Despite these research advances and efforts by the National Institutes of Health (NIH) to involve ANAI people in major precision medicine programs, many ANAI communities remain hesitant to participate in precision medicine due to concerns about data management and other aspects of precision medicine (Hiratsuka et al., 2012a,b; Avey et al., 2016; Tsosie et al., 2019; Beans et al., 2020). Precision medicine involves large datasets and extensive data-sharing, features that increase privacy risks and undermine the ability of participating individuals and communities to control who can access their data and for what purposes it is used. For Tribal communities, the terms of data management in research and clinical care are entangled with Tribal sovereignty and the inherent rights of self-determination and self-governance. Approaches to data management that limit ANAI ownership of, control over, access to, and possession of data not only endanger individual autonomy and expose participants to indeterminate privacy risks, they also, and uniquely for Tribal communities, are challenges to fundamental political rights (Warren-Mears, 2012; Tsosie et al., 2019; Woodbury et al., 2019a). Mistrust engendered by perpetration of unethical research on ANAI people has only sharpened the questions these communities raise about data management and the conduct and goals of research more broadly (Mello and Wolf, 2010; Pacheco et al., 2013; Beans et al., 2019).

### Health Communication

Tension between the potential for ANAI people to benefit from precision medicine and the hesitancy among some ANAI communities to engage as patients or participants in this approach to clinical care and research underscores the need to develop ways to communicate clearly and accurately about precision medicine. Such messaging would provide ANAI individuals, communities, and organizations with the information required to make determinations about whether and how to implement, utilize, or participate in precision medicine programs. Tools and conceptual models developed within the communication field are uniquely well-suited to addressing this informational need.

Health communication is variously defined as the “the study and use of communication strategies to inform and influence decisions and actions to improve health” (Making Health Communication Programs Work, n.d.) or “any type of human communication whose content is concerned with health (Rogers, 1996, pg. 15).” Health communication is increasingly recognized as indispensable for promoting health literacy and informed health care decision-making. Academic institutions, government agencies, non-profit health policy institutes, and professional organizations have devoted resources to health communication research and education and national-level public health initiatives include health communication objectives (Institute of Medicine (IOM), 2002; ODPHP (Office of Disease Prevention Health Promotion), 2014; APHA (American Public Health Association), 2019; Harvard, 2019).

**FIGURE 1 |:**
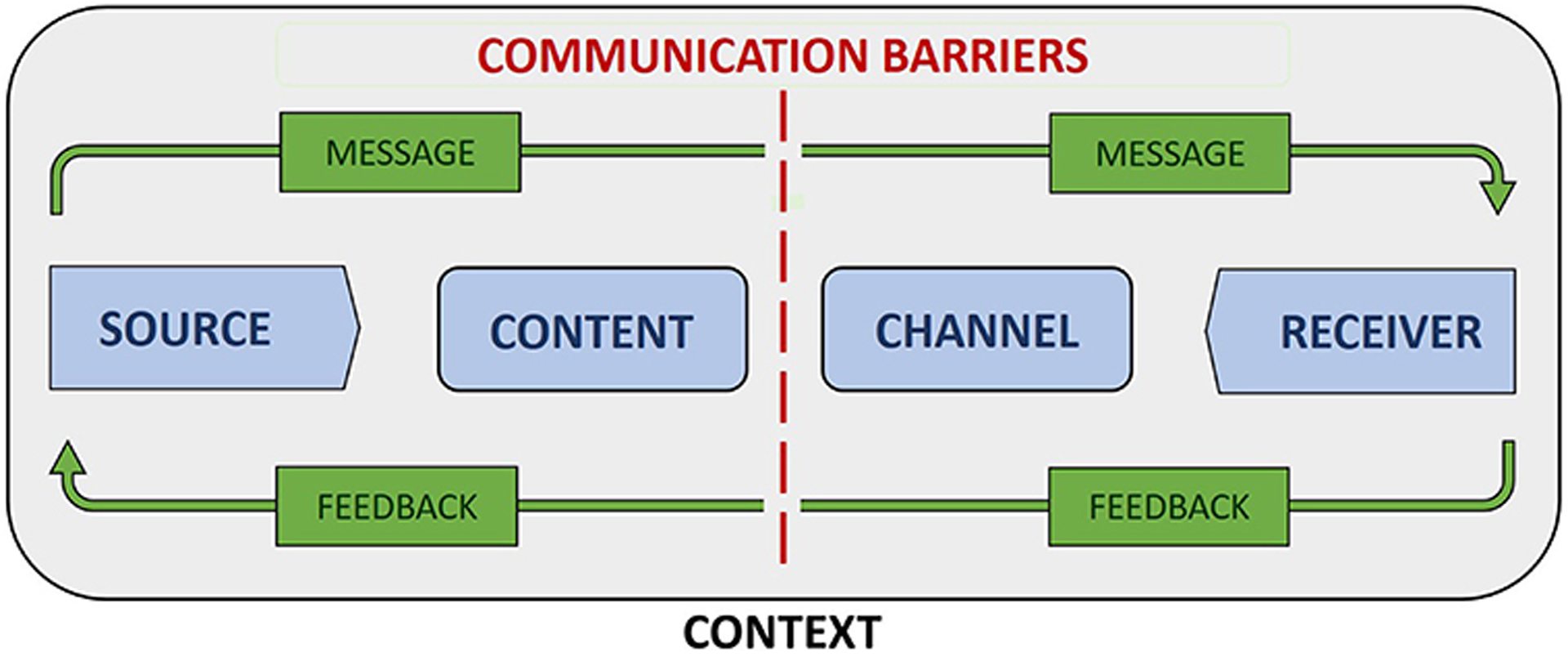
General communication model.

Figure 1 depicts a general model of communication that combines elements of early linear models of communication (Berlo, 1960; Shannon and Weaver, 1963) with those from subsequently developed interactional models (Schramm, 1955) and transactional models (Barnlund, 1970). Model components include source and receiver, message content and channel, context, communication barriers, and feedback. Source and receiver respectively refer to the transmitter and recipient of a message (West and Turner, 2018, pg. 9). In health care settings, a provider or health care system is often the source and a patient or patient population the receiver of health information. Message content refers to the actual information contained in a communication, while message channel refers to the medium (e.g., verbal speech, written text, visual symbols) or sensory pathway (e.g., sight, hearing) by which information is passed from sender to receiver (West and Turner, 2018, pg. 9).

Context refers to the range of physical, psychological, social, cultural, and relational factors that shape communication processes (Institute of Medicine (IOM), 2002, pg. 29). For example, the availability of communication resources within an organization is a contextual factor that determines the possible approaches to communication, including the frequency, quality, and variety of messages and the channels by which messages can be transmitted. Culturally-informed communication preferences are contextual factors that impact the acceptability of different methods of information delivery, such as educational posters and brochures, discussion groups and patient-provider encounters, or social media and email. Background noise in a clinical waiting room and a patient’s emotional state and perspective on medicine are physical and psychological factors affecting transmission and receipt of health communication.

Communication barriers, often referred to as “noise” within the communication literature, are those physical, psychological, physiological, and semantic factors that negatively impact the accurate and complete transmission of information between source and recipient (West and Turner, 2018, pg. 10). Examples of noise include language barriers, social norms that affect the interpretation of specific terms and concepts, physical and physiological barriers—such as unreliable internet access and hearing impairment—that interfere in the transmission and receipt of messages, errors of syntax that affect the clarity of a message, and loud or distracting environments. By identifying the sources and extent of noise, individuals and organizations are better positioned to develop communication plans that limit the negative impacts of noise on the accurate and complete transmission of health messages.

Interactional models of communication incorporate feedback in recognition of the fact that communication is often bidirectional in nature, in that recipients both receive and respond to messages received from sources (West and Turner, 2018, pg. 11). Examples of feedback include a recipient repeating the message back to the source or asking clarifying questions. Feedback can provide confirmation that a message was received and inform changes that improve message effectiveness as measured by its ability to progress toward a specified goal. Incorporating feedback into communication models can help prompt development of communication plans that include processes designed to encourage and react to recipient responses.

The generalized nature of this model makes it applicable to a broad range of communication types and contexts. It also has its roots in communication theory rather than social or health marketing, in which information is often provided in a manner designed to persuade individuals to adopt new health beliefs and behaviors (CDC, 2020). While appropriate in the context of public health programs with marked, near-certain benefits and minimal to zero known risks, persuasion can easily shade into manipulation and other species of influence that are ethically questionable or impermissible in health research or in novel clinical interventions (Powers, 2007; Blumenthal-Barby, 2012). By contrast, Figure 1 depicts a model concerned with the objective transmission of information, making it well-suited for conceptualizing potential approaches to communicating about precision medicine.

### Challenges for Communicating About Precision Medicine

Effective health communication is crucial to the successful implementation of any novel clinical service or research endeavor but may be especially important for precision medicine, due to its complex and controversial nature. However, those factors that suggest the need for a considered approach to health communication are also the source of communication challenges. In the case of precision medicine, several factors make it difficult to fully and accurately describe what health systems and individuals are committing to when they implement or participate in these programs.

First, informed decision-making regarding involvement in clinical or research applications of precision medicine requires familiarity with concepts that, due to their novelty and/or complexity, are inherently difficult to explain or are prone to misinterpretation. For example, genetically-based disease risk is often uncertain and probabilistic in nature, rather than certain and deterministic (National Academies of Sciences Engineering and Medicine, 2016). Low levels of numeracy and health literacy in the U.S. general population only add to the difficulties inherent in communicating about these kinds of risk (Berkman et al., 2011). Similarly, lay intuitions about genetics are prone to essentialism and exceptionalism, exaggerating the causal and deterministic role of genes in health outcomes and the unique status of genetic information (Garrison et al., 2019; Heine et al., 2019). Each of these topics is integral to precision medicine and all pose communication challenges. Second, precision medicine is distinct from other approaches to clinical care and research in the extent and variety of data it collects from, and the demands it may place upon, research participants. As a result, participation in precision medicine research will likely involve increased risks of privacy loss and subsequent harms and require an uncommon level of commitment from participants (Wagner et al., 2017). In order to communicate effectively about the practical and ethical implications of these differences, researchers will need to develop new ways of speaking about the risks and burdens of participation. Similarly, where precision medicine research efforts involve use of broad informed consent, researchers will need to find ways to speak cogently about research goals and data uses that cannot be fully specified. Other communication challenges include managing public perceptions of precision medicine and enabling exchange across disciplinary lines. Depictions of precision medicine have been criticized as overpromising on potential population health benefits, highlighting need for balanced messaging that is neither overly optimistic nor unduly skeptical (Duffy, 2016; Maughan, 2017). The inherently multidisciplinary nature of precision medicine will require providers and researchers working with different conceptual frameworks to develop a shared language for communicating about precision medicine (Scherr et al., 2017).

There are additional challenges specific to communicating with ANAI people about precision medicine. As alluded to above, some ANAI communities have been exploited by biomedical researchers and are consequently hesitant to participate in research, particularly when research involves privacy risks, broad data access, and unclear benefits for individuals and communities participating in research. Scholars have noted that precision medicine requires considerable trust in precisely those health care and research institutions that ANAI people and other minority groups are justifiably mistrustful of (Christopher et al., 2008; Pacheco et al., 2013; National Academies of Sciences Engineering and Medicine, 2016, pg. 7). Any effort to communicate about precision medicine within a Tribal context will have to account for these concerns. Additionally, some aspects of communication norms that are characteristic of ANAI people are distinct from those found in other populations. These norms not only impact the language and terminology most appropriate for use in a given community, but also affect preferences regarding communication context, mode, source, and audience (Satter et al., 2005; Woodbury et al., 2019b). Finally, there are challenges specific to the health care systems that serve ANAI people. Tribal health care systems are chronically underfunded and provide care to both urban populations and extremely remote communities spread over a large geographic area (Sherry, 2004; Warne and Frizzell, 2014; United States Commission on Civil Rights, 2018). Additionally, some systems have adopted unique, culturally-informed approaches to patient care that affect patient and provider expectations related to interpersonal communication (Gottlieb, 2013). Health communication strategies employed within these systems will need to consider the cost-effectiveness of different communication tools, be applicable across a range of patient groups and care settings, and be tailored to institutionally-specific communication approaches.

Research describing efforts to address these barriers as part of larger strategies for implementing precision medicine programs within health care organizations is extremely limited, despite calls from communication scholars to undertake such investigations (Scherr et al., 2017). More focused research that explores how research and clinical applications of precision medicine should be communicated to ANAI communities and the health care systems that serve them is also lacking. The need for such research will only grow as precision medicine transitions from an emergent to a mainstream approach in research and clinical care and ANAI communities and individuals are increasingly faced with decisions about whether and how to engage with precision medicine programs. In an effort to address this need, the present study characterizes patient, provider, and leader preferences on communicating about precision medicine in the context of a Tribal health care system. The study responds to the following related research questions:

What information do patients, providers, and leaders need in order to make informed decisions about whether to participate in precision medicine research, utilize clinical applications of precision medicine, and implement a precision medicine program within a health care system serving AN people?

With respect to information sources and audiences, message channels, communication barriers, and relevant contextual factors, how should this information be shared?

## MATERIALS AND METHODS

### Study Setting

Interviews took place at facilities operated by Southcentral Foundation (SCF), a non-profit Tribal health organization headquartered in Anchorage, Alaska that provides a wide range of primary and specialty health care services to over 65,000 ANAI people. SCF is nationally recognized for its Nuka System of Care, a culturally-grounded, patient-centered, and relationship-based approach to primary care that emphasizes and supports communication between patients and providers [Gottlieb, 2013; NIST (National Institute of Standards Technology), 2017]. SCF’s Anchorage Native Primary Care Center has seven primary care clinics, as well as pediatric and women’s health clinics. The primary care clinics are staffed by integrated care teams that include a primary care provider, certified medical assistant, scheduler, behavioral health consultant, nutritionist, pharmacist, and registered nurse case manager (Driscoll et al., 2013). Each patient is empaneled to an integrated care team and family members are encouraged to receive care from the same team (Driscoll et al., 2013). A majority of SCF’s employees are of ANAI heritage and are eligible to receive health care services at SCF [NIST (National Institute of Standards Technology), 2017].

Research pursued by SCF is determined by Tribal health priorities and informed by the principles of community-based participatory research (Hiratsuka et al., 2017a). All studies conducted by SCF staff and/or with the SCF patient population have received approval from the various SCF Research Review Committees (Hiratsuka et al., 2017a).

The Alaska Area Institutional Review Board and SCF Research Review Committees and Executive Board approved this study protocol and manuscript prior to journal submission.

### Research Participants

Research participants were members of 3 stakeholder groups: (a) adult patients of ANAI heritage seen in SCF primary care at least once in the last 3 years; (b) primary care providers or providers who receive SCF specialty care referrals; and, (c) Tribal health care system leaders who direct primary care activities and/or health research at SCF. Providers included physicians, nurse practitioners, behavioral health consultants, pharmacists, and a dietician. Leaders included medical and administrative directors and division vice presidents.

### Recruitment

Patients were recruited in the lobby of the SCF Anchorage Native Primary Care Center. Providers and leaders were recruited by email. All potential participants were screened for eligibility. Individuals who met inclusion/exclusion criteria were scheduled for an interview with a research team member. Patients received a $25 incentive for participating in the study; providers and leaders were not compensated for their time.

### Data Collection

Interviews were conducted between January and August 2017 by research staff trained in qualitative methods (VH, JB, and RW). Verbal informed consent was obtained from each participant prior to the start of the interview. We used verbal consent to minimize confidentiality and privacy risk for participants. Interviews lasted ~45–60 min. A semi-structured interview guide was used for each interview. The guide included a definition of precision medicine and questions on communication needs and preferences. Interviewers also asked questions about the National Institute of Health’s (NIH’s) *All of Us* program, using an NIH infographic describing the program as a show card. See Supplementary Materials for the interview guide and (Beans et al., 2020) for further details on the showcard. Personal identifiable information was not collected. Interviews were audio-recorded and transcribed verbatim. Interview data was stored on a secure network server only accessible by password-protected computers used by SCF staff.

### Data Analysis

Transcripts were coded using Atlas.ti 8.3.20.0 (Scientific Software Development GmbH, Berlin, Germany). Thematic analysis was used to analyze these data through an iterative and inductive process (Attride-Stirling, 2001). A priori codes were derived from constructs represented in the interview guide. A sentinel transcript from each participant group was coded by four members of the research team (VH, JB, RW, and KW) to refine code definitions and increase inter-coder reliability. Subsequent transcripts were coded by a research team member and then reviewed by a second research team member. See Beans et al. (2020) for a description of codes used in the initial analysis.

For this study, we identified codes describing topics relevant to communicating about precision medicine, including: return of results; management of patient response to return of results; communicating about risk/probability; perceptions of genes and genetics; and, health care system resources to aid return of results. Quotes linked to these codes were reviewed and sorted according to their pertinence to the research questions. Pertinent quotes were then organized into themes corresponding to the components of the general model of communication depicted in Figure 1.

## RESULTS

### Results Overview

A total of 46 interviews were conducted with patients (*n* = 21), health care providers (*n* = 12), and health system leaders (*n* = 13). Participants described their perspectives on precision medicine and potential approaches to implementing a precision medicine program at SCF. Recommendations for communicating about precision medicine to patients and providers figured prominently in these accounts. Participants rarely distinguished between the research and clinical applications of precision medicine. For this reason, the results do not make this distinction except where allowed by explicit participant comments. Results are organized according to the components of the communication model described above.

### Communication Sources and Recipients

Patients, providers, and leaders were all identified as both sources and recipients of information about precision medicine. However, patients were understood to be the primary recipients and providers the primary sources of information about precision medicine.

A provider thought behavioral health consultants were experienced at discussing difficult issues with patients and the interpersonal communication skills cultivated through these encounters would leave them well-prepared to discuss precision medicine with patients who had concerns about research consequent to experiences of unethical research and mistrust of government. One leader preferred to receive information about precision medicine from their primary care provider, noting that their long-term relationship with this individual made it easier to discuss options like precision medicine that involved benefits and risks.

Leader: “I already have a relationship with my primary care provider… I don’t have the same relationship with the CMA [certified medical assistant], so if there was a new type of blood test that was going to help inform my health, I would probably feel most comfortable if it came from my primary care provider that I already know and it’s already in relationship with… And I think that I would want to hear from my primary care provider on what would be the benefits and risks associated with this and what are we going to learn from that, and how is that going to help me on my wellness journey? I don’t think I would take it in as much from the CMA that I’m seeing, because I don’t—every time I go in, I have a new CMA… So, I think it depends on that relationship.”

Recognizing the role providers play as sources of health information, participants also proposed that providers receive essential information about precision medicine through formal training or educational opportunities. For example, several participants suggested in-person or online trainings that would enable providers to answer patient questions and encourage discussion around precision medicine.

Provider: “Encourage people to have discussions with their primary care providers… while primary care docs, especially, should be educated on just what [precision medicine] means. ‘How do I—how do I understand it? What’s the downside?…What are the false positives and false negatives and the ramifications to that?’ But just to allow conversation… Anything that promotes discussion and lets people be curious and lets people voice their concerns, I think, is a reasonable approach moving forward.”

Leaders were described as spokespersons who could articulate the organization’s stance on precision medicine. One patient noted that public support for precision medicine among Tribal leaders would help reassure patients and providers of the legitimacy of precision medicine as a safe and potentially beneficial approach to clinical care and research. It was also recognized that leaders, although not requiring the kind of detailed knowledge of precision medicine expected of providers, should be familiar enough with the potential risks, benefits, costs, and operational details of precision medicine to make decisions about whether and how to make precision medicine available in clinical settings or to undertake precision medicine research.

Patients also viewed one another as potential sources of information about precision medicine. Participants noted that parents have experience at and a vested interest in educating their children, making them a natural choice for communicating to younger generations about precision medicine. Patients also indicated a preference for learning about precision medicine via discussion groups where they could benefit from exposure to the knowledge and questions of other patients. One patient stated that positive experiences with precision medicine would leave them able to serve as advocates for and sources of information about precision medicine. Participants saw patients as needing the kinds of information (described in the following section) that would enable them to make informed decisions about whether to participate in precision medicine research or utilize clinical applications of precision medicine.

Patient: “And if it works for me, I can point [to] my other nephews and cousins and everybody else and say: ‘Go try this out. Go see these people.’… Because I want to be a spokesman and I will say, you know, ‘This is what works. This is how I combat this or that.’ And I’d have an avenue to say, “Hey, go try that program out.”

Finally, participants identified the broader community as recipients and subject matter experts as sources information about precision medicine. Leaders and providers raised the possibility of training or hiring staff to function as subject matter experts who could provide on-demand support for providers and patients requiring information about precision medicine. Incorporating this service into SCF’s Nuka system of care was suggested as a means to aid decision-making while saving patients and the health care system the time and costs of an additional specialty consult.

Leader: “So, one of the things that we seem to do well here are having [subject matter experts], right?… If there was somebody identified to go to who really knew the ins and outs… somebody that’s very, very knowledgeable, who’s available for questions over time. Because there’s always going to be: ‘This is something I did not anticipate, I have no idea how to respond to this.’ So [providers] are going to need somebody to go to.”

### Communication Content

Participants described the information ANAI people should be given when asked to participate in precision medicine or being approached about precision medicine. This included a definition and basic description of precision medicine; the potential benefits and harms of participating in or implementing a precision medicine clinical or research program; the costs and other impacts of implementing precision medicine within the Tribal health care system; and, a review of research goals and processes and the terms of data management. Participants also recognized that informational needs varied across stakeholder groups and described group-specific communication content.

#### Definition of Precision Medicine

Members of all participant groups stated they would require a definition or description of precision medicine in order to make an informed decision about participation. Several participants added that accounts of precision medicine would need to be comprehensive and that they sought to fully understand what precision medicine is and what it means for patients and other stakeholders. Requested information included the types, intended use, and potential health benefits and risks of any tests involved in precision medicine. This information was characterized as the “who, the what, the how” of precision medicine. One provider noted that possessing this knowledge enabled individuals in clinical or teaching roles to respond adequately to questions posed by patients.

Provider: “In terms of getting the information out to Alaskan Native people, just providing this in a very clear manner about what it is, what it means, what it can do for our system, what it can do for them individually/So, I think that, again, transparency is really huge.”

#### Benefits and Harms of Precision Medicine

Providers, leaders, and patients emphasized the need for information about the potential benefits and harms of precision medicine. One provider stated that the benefits and risks of precision medicine for future generations would strongly influence patient perspectives and decisions related to these programs. A leader stated that patients would need to be informed about short-term, personal benefits of participation and whether longer-term, group-level benefits were possible. Participants named specific benefits and harms that they expected to be associated with precision medicine and that they would want to have information on when deciding whether to become involved in clinical or research applications of precision medicine. Notably, potential harms of precision medicine were discussed at greater length and by more participants than were potential benefits.

Identified benefits included the potential to improve the safety and effectiveness of treatment plans by enabling clinicians to account for and adapt to individual-level differences in medication response. One provider suggested that precision medicine could increase the efficiency of the process for selecting and prescribing medications by replacing a largely trial and error approach with the ability to quickly identify appropriate, targeted medications. Providers and leaders thought that findings from precision medicine would be particularly useful in guiding selection of treatments for patients with diseases that are difficult to treat or diagnose, including cancer, chronic pain, and complex childhood illnesses.

Provider: “I think [patients] would really love it. I think a lot of people would be interested in having that kind of assessment and plan tailored specifically to what’s unique about them… I’ve heard about it being used for chronic pain and the differences in efficacy for different pain medications addressing pain and I think it might be useful in that. Not just chronic pain, but pain in general… I guess when it comes to disease prevention, just having a better, a clearer idea of what someone might be predisposed to.”

Participants from all groups noted that that the SCF patient population is heterogenous and comprised of multiple cultural groups that are distinct from one another and from the general population in numerous ways. One patient was excited about the possibility of leveraging precision medicine to characterize these differences and then using this information to drive treatment decisions. In related comments, participants spoke to the importance of involving ANAI people in large-scale health studies to ensure that research findings were relevant for this population and a provider advocated that SCF implement a precision medicine program for the same reason.

Leader: “I think that a lot of times American Indians, Alaska Natives are—research isn’t done, in terms of health. And so a lot of times you have to extrapolate from non-American Indians and Alaska Natives and apply it to American Indians and Alaska Natives, and that isn’t the best way to do it, because there’s differences in the populations, as we know. There’s even differences within that population of American Indians and Alaska Natives itself, so you’re also extrapolating within that population and making assumptions that may or may not be true. But if you at least have that group of people, enough of those people included in the study, then you can actually make some decisions that make sense.”

Patient: “Are there differences between Alaska Natives versus the rest of the population? It’s a huge population. Let’s break it down. Let’s take a look at the differences of what’s going on in the environment. If there are findings that are different in Alaska Natives versus the rest of the population, what can we do? Is it positive? Is it negative? Let’s take a look at that. Let’s adapt. Let’s see how we can improve our health across the board.”

Potential benefits related to diet, exercise, and disease risk information were also discussed. One patient appreciated the holism of precision medicine in that it considered the impacts of environment, diet, and lifestyle on individual health. Another noted that mainstream nutrition guidelines and recommendations do not speak to traditional subsistence diets and wondered whether precision medicine could provide ANAI people with guidance on how to maintain a diet that is both healthy and traditional. A third patient appreciated that precision medicine would enable individuals to be informed about their risk for chronic diseases, including those that were highly prevalent in their communities.

Patient: “I’m half Navajo and everybody on the Navajo side developed diabetes. Whether they were exercising frantically every day, they still developed diabetes by the age of 45 or 40. I didn’t get it till 45. [ANAI people] need to know things like that, what could happen… the propensity for them developing diabetes or heart disease or any other malady that may occur later on in life. That’s important for them to know.”

Identified harms included the potential for precision medicine to lead to discovery of genotype-phenotype associations that reinforced negative stereotypes about ANAI people and for genetic testing to undermine individual and familial identities or community ties if test results challenged an individual’s status as a member of a family or group. Participants expressed related concerns about the potential for genetic test results that revealed unexpected paternity information to negatively impact relations among family members.

Provider: “Well [precision medicine] has gotten the same kinds of concerns and fears… that if Alaska Natives or American Indians are discovered to have a certain genetic mutation in higher frequency than the general population, then what kind of discrimination and prejudice will be held by the people who don’t have that or the people that make policies or are determining how the money is being spent, what kind of prejudices will come up?”

Leaders and providers also worried about the potential negative impacts of disease risk information on the cost of health insurance and employment opportunities. For example, one provider wondered whether an identified genetic risk for disease would lead to higher health insurance premiums, even in the absence of a formal diagnosis, symptoms, or need for treatment.

Leader: “Once you find out and have someone’s DNA and you start looking at family history and then start projecting they could get this or that, I don’t know that that’s a good benefit. And then I don’t know the impact of—is that going to impact their ability to get insurance?”

Provider: “I wonder if it would ever hurt somebody to know that they may be predisposed to something… If that information was outside of our system, would it prevent them from possibly being covered by insurances or anything like that?”

Patients and leaders expressed concern about the potential for precision medicine to promote deterministic conceptions of the role of genes in health that discouraged individuals from taking ownership of their health. One leader openly questioned the extent to which health was influenced by genetic factors and worried that an undue emphasis on genes would distract away from crucially important environmental and social determinants of health.

Leader: “I’m not sure how much of our wellness is impacted by our genetics that we start with compared to our lifestyle and behaviors. I think understanding our genetics and using that to better deliver health care is important but I wouldn’t want it to overshadow our behaviors and our lifestyles: what we eat, what we drink, how we parent, how we were parented, how we handle stress, how we handle anger, how we feel about ourselves, our self-confidence, our level of value that we place on ourselves, our level of empowerment. Those for me are huge drivers of multidimensional wellness for people, families, and communities.”

Participants also worried that the potential for precision medicine to identify an optimally effective or cost-effective intervention or treatment plan could have the perverse effect of limiting patient choice, since alternatives would be seen as less desirable from a value or effectiveness standpoint. Similarly, one leader wondered if patients would experience negative psychological impacts if a purportedly optimal treatment turned out to be ineffective. Another participant worried that precision medicine would cause health care systems to take an authoritarian turn and pressure patients to submit to specific treatments because of their cost-effectiveness.

Leader: “I think the drawback or risk might be providers and/or [patients] kind of feeling boxed into a particular type of intervention. So, if you were able to look at, for example, this medication would help you and this medication would not help you, would that create a little bit of ‘all or nothing’ thinking? And could that have potential downsides for providers not exploring other things or kind of getting locked into that area… If [patients] were solely focused on this intervention and found out that its actually not going to be that helpful for them, could it initially—could they lose hope?”

Participants noted the potential for the kinds of unforeseeable harms that arise from technological and scientific advances and for harms that were known but were contingent upon factors—such as political climate and federal data management policies—that could not be controlled or accurately predicted. One leader was hopeful but uncertain that past research abuses would not be repeated and expressed a need for information about participant protections.

Provider: “If you’re identified as a certain kind of individual, then you can be treated differently, in a negative way. So that’s the main thing that anyone including myself is concerned about. Will you have to pay more for your insurance? Will you be treated negatively by your own team because they have prejudice against you for having some kind of [genetic] mutation?…it’s pretty unlikely that those things are going to happen. But people naturally worry about those things because they’re worried about prejudice.”

Leader: “And so, I feel like there are safeguards in place to prevent anything really bad from happening. Like, Tuskegee isn’t going to happen again, right—hopefully, right? So, I’m conflicted. I really am conflicted. I would need to be informed on the safeguards, I guess, so that would be important.”

A majority of participants identified both benefits and risks to participating in precision medicine but few spoke to whether precision medicine was likely to have an overall positive or negative impact on the patient population served by SCF. Participants expressed a guarded, conditional optimism about the potential benefits of precision medicine but also spoke candidly about past research abuses perpetrated upon ANAI people and their concern that precision medicine would recapitulate this history.

Patient: “While I am hopeful and can see the benefits, I worry that it goes wrong… I think that, while this has lots of potential for good, it’s also got the potential to go awry.”

Patient: The downside is, anytime you get that kind of information, it depends on the security of that information, because it always depends on what you are pulling the information for and how it is being used and if it could be misused…Other than that, I think it’s great. It’s just—there is always the dark side everything.

Patient: “There has been some mistrust in the past about what has transpired with Alaska Natives across the board because of research in the past, historically without consent, without understanding. I think that would be my only concern. So long as that’s not violated and someone is made to understand, then I’m all for it.”

#### Health System Impacts and Costs of Precision Medicine

Participants described the need for information on how precision medicine would impact clinical care. For example, providers wondered how precision medicine would be integrated into existing services, what sorts of clinical actions would be triggered by test results that indicated a patient was at risk for developing an inheritable disease, and whether results from genetic testing conducted as part of a precision medicine program would substantively change individual- and population-level interventions. Participants also emphasized that any clinical impacts would need to be described in concrete terms: one patient expected to be informed on how precision medicine would improve mental and behavioral health care and a provider wanted case examples of how precision medicine could be employed in psychiatry.

Participants also raised questions related to the costs and value of precision medicine and thought this information would be crucial for determining whether and how precision medicine should be implemented at SCF. For example, leaders and providers raised questions concerning the affordability of precision medicine for patients and whether Medicaid/Medicare would cover the costs of clinical services; the financial sustainability of precision medicine programs; and, whether investing in precision medicine would divert resources from services with known population health benefits. Participants also desired information on how precision medicine would add value to the range of services already available at SCF and some questioned whether results from precision medicine tests would significantly alter clinical practices.

Provider: “How is this project going to be different from what they can already get? And is there a big enough difference that there would be enough motivation to do this research project? Because if they’re going to come out saying, ‘Yes. You have 90 percent chance of Type II diabetes if you gain another 20 pounds.’ Well, we already know that. We don’t need research for that. So how would genetic testing knowing that you have 12 family members with Type II diabetes going to help you any more than what we already know?”

#### Data Management

For both clinical and research applications of precision medicine, having information about the terms of data management was considered essential by all participant groups. Patients, providers, and leaders providers stated that they would want information on several components and principles of data management, including with whom and for what purposes data would be shared; who would have access to data and the conditions under which it would be stored; how data would be analyzed and interpreted; what entities would retain control over and ownership of data; whether data would be identifiable; and, what protections would be in place to ensure participant privacy.

Patient: “I would want to know: Is my identity protected? Who would have access to my individual information?…The third thing is: What is it going to be used for? And I think that would be it. I think that would be just the three things I would most be concerned about. You know, having my identity protected.”

#### Research Project Information

Patients, providers, and leaders expected that participants would want to be informed about the time commitments required of participants involved in precision medicine research projects, the nature of any project procedures, and whether they would have access to research findings. Patients also asked for clarity about research goals, progress, and outcomes and expected to be informed if adverse events occurred. One provider stated that for any precision medicine study they would expect to be informed about research goals, timelines, Tribal support, and safety and ethical issues, as well as information about the sensitivity and specificity of genetic tests.

Provider: What does it take—what do I have to give up? Do you stick a needle in my arm, a blood sample or do you stick a needle in my spine? What does it actually involve, and who’s going to see the information? What—who has access to the information?

#### Group-Specific Informational Needs

Participants recognized that different individuals and stakeholder groups at SCF had varied informational needs in relation to precision medicine and described communication strategies that accounted for this diversity.

For example, participants thought that leaders needed to be able to decide whether and how to implement precision medicine at SCF. If a decision was made to pursue implementation, leaders must also be prepared to offer informed and visible support of precision medicine. To this end, one participant suggested that leaders would need information that allowed them to determine the extent to which precision medicine could be aligned with SCF’s operational principles and another stated that leaders required a discussion-oriented and non-technical introduction to precision medicine that enabled them to respond to questions and concerns raised by community members.

By contrast, participants thought that providers needed to have a basic knowledge of precision medicine and be able to answer common questions about this area of research; to understand how precision medicine might affect clinical practice; and, to be able to translate core precision medicine concepts into layman’s terms.

Finally, participants thought that patients needed information that enabled them to make informed decisions regarding involvement in clinical or research applications of precision medicine. Given SCF’s commitment to involving patients in the identification of organizational health and research priorities, participants also suggested that patients be provided with the information they needed to contribute to discussions concerning the implementation and structure of precision medicine programs. Participants recognized that different patient groups would have varied informational needs and that messaging about precision medicine would need to account for this diversity. Factors including age, health literacy, and family planning were thought to influence patient informational needs. In particular, several participants noted that Elders may be less familiar with concepts and terms relevant to precision medicine and would therefore require more background information than younger patients with greater exposure to these concepts and terms.

### Communication Channels

Participants identified several channels for communicating with patients, providers, and leaders about precision medicine.

Participants frequently suggested the use of brochures, posters, and video messaging to share information about precision medicine with patients. Several participants remarked that such tools were often used to great effect as part of SCF’s health communication and promotion efforts. Providers and leaders noted that posters placed inside exam rooms helped spark conversation with patients in a safe and private setting. Participants appreciated that brochures, flyers, and pamphlets could be placed around campus and easily distributed to patients. Patients, providers, and leaders all recommended use of the passive education panels (PEP)—video monitors placed in primary care clinic waiting areas and used for public health and health services messaging—to disseminate information about precision medicine.

Leader: “Posters in the [patient examination] rooms are great… People like the messaging on the TV’s. People will often ask me about those things. Like, ‘Hey, I saw that on the TV while I was waiting. Can you give me some more information about that?’ So that’s always a good conversation starter.”

Members of all participant groups emphasized the importance of including person-to-person communication methods as part of a precision medicine communication strategy. Recommended methods included discussions with providers during wellness visits and other clinical appointments, focus groups and learning circles (i.e., small discussion groups offering patient education and support), and large community events. Those in favor of learning circles and other small group discussion formats highlighted the benefit for individuals of being exposed to a range of novel ideas, questions, and perspectives. Participants also noted that learning circles could be readily focused on topics of interest to discussants—such as the role of precision medicine in the management of specific diseases or responding to precision medicine results—and that the shared interests and experiences of individuals attending such groups would be a welcome source of empathy. One participant proposed small group presentations where individuals who had participated in precision medicine could report on their experiences and where the potential outcomes of participation in precision medicine could be explored. Finally, participants recommended hosting informational booths or sessions at health fairs or large public events that are attended by ANAI people from across the state.

Patient: “When you do it in group settings, what’s nice is that we all think differently. When you get a group together and you collaborate, you get different insights and views in small little glimpses that you would’ve never thought about your own because we all think differently. I think that would be extremely helpful because then that way you to… make a better decision on how you want it to affect you or how you want to be a part of it.”

A majority of participants suggested more than one channel for communicating about precision medicine and several explicitly stated that a multiplatform approach to communication was needed. A provider and leader justified the use of several communication methods by noting that there is no single, monolithic learning style and that providers needed to have access to a range of informational resources in order to meet the needs of patients with a variety of learning styles and preferences.

Leader: “I think that you have to do it 100 different ways… I think that we would have to have some focus around it at the annual gathering where the whole community is invited. We would have to put it in our newspaper, we would have to put up posters, we would have to put it in the PEPs [passive education panels] that are in… the waiting areas. We would have to have flyers, we would have to have scripting.”

Participants thought that the communication channels used to disseminate information would need to be tailored to the preferences of different patient groups. Patient age was the factor most often identified as affecting these preferences. For example, although participants did not recommend the broader use of social media platforms, websites, email, and other digital applications to share information about precision medicine, these communication channels were described as being appropriate for outreaching to younger patients due to their greater familiarity with computers and the internet. By contrast, Elders were described as preferring more traditional methods of disseminating health information, including educational posters and in-person discussions with providers. Middle-aged and older adults were thought to use both of these types of communication channels.

Leader: “I would say that the newspaper would be for my mother’s generation… but for the younger generation, it would have to be on the phone, it would have to be mobile, it would have to be Instagram, it would have to be—you know, Snapchat… I mean, so I think that it would be definitely more technology based with the younger generation. And I think that, for—you know, it would probably be a mix for people who are in their 40s. There may be some—or 50s—there may be some who still like the paper and the printout and letters and there’d be some who have it all electronic, so I think it would be a mix for me.”

### Communication Context

Participants described how cultural and institutional context impacted the acceptability and effectiveness of health communication efforts, and proposed steps to account for these factors. These actions included adapting the terminology used to describe precision medicine to account for the history and linguistic and cultural norms of the patient population and aligning the approach to communicating about precision medicine with culturally relevant guiding principles.

Participants identified specific terms used to describe major precision medicine initiatives that were unlikely to be well-received by patients. “Genetics” and “genetic testing” were terms that participants described as “technical,” “scary,” and “invasive” and thought would be meet with suspicion, fear, or confusion by some patients. “Precision” and “Precision medicine” also troubled participants, who found the term unfamiliar, mechanical, and suggestive of Western approaches to medicine and science. Other words, such as “tell,” “engage,” “research,” and “experimental” that might seem innocuous to researchers also raised reg flags for participants. For one provider, the word “apply” used in the context of a discussion about precision medicine evoked images of guinea pigs in a laboratory—of research subjects rather than participants or partners. This provider explained that fearful or otherwise negative reactions to these terms, which might seem exciting or simply unremarkable to researchers, were a response to ANAI people’s experiences of unethical practices. Another provider felt it was important to clarify that precision medicine was not an “experimental” treatment and recommended describing precision medicine in terms of an emerging approach to research that was actively being implemented in other leading health care systems in the United States.

Provider: “I think that one of our greatest learning moments at SCF as we’ve designed our system is to understand the value of language and of having shared language and shared understanding as a community for things. When we say ‘customer-owner’, we want everyone to understand what we mean by that term and what value it holds. I think the word ‘precision medicine’ does not hold inherent meaning in those words. Nobody is going to understand what you’re talking about when you say precision medicine. It sounds like a surgery word or something.”

Leaders and providers were also clear that developing culturally appropriate approaches to communication involves more than adapting terminology to account for local preferences. One leader spoke of the need to frame precision medicine in terms of how it complements the existing research portfolio at SCF and its alignment with the mission, vision, and values of SCF. In sharing results of precision medicine with the ANAI community and the broader public, providers and leaders advocated using a strengths-based approach in characterizing population-level epidemiological trends. For example, one leader suggested that informational materials about and results from precision medicine could emphasize cultural resiliency even in the context of descriptions of elevated disease risk and another leader recommended avoiding generalizations that could introduce or contribute to negative stereotypes about ANAI people.

Leader: “I think it has to always celebrate the strengths and the resiliency of the people, not just show the negative. For example, if you’re talking about how there’s an increase in childhood disease or how there’s an increase in cancer amongst our people, we want to understand how the strengths of subsistence, how the healthy foods of our people can help combat this. So that you’re celebrating these strengths and seeing them as a solution.”

Similar emphasis was placed on the need for an approach to communication guided by the ethical principles of transparency and respect for the autonomy of patients and participants. Several participants stated that descriptions of the potential benefits and harms of precision medicine should be transparent and balanced. Informational materials should present any information known to be relevant to individual- and community-level decision-making. Although participants concurred that transparency required provision of information on precision medicine, they were divided over how to uphold this principle without overloading patients with unnecessary information. Regarding respect for persons, participants thought it crucial that descriptions of precision medicine emphasize the voluntary nature of involvement in clinical and research applications of precision medicine. Providers should present precision medicine as an option or opportunity rather than as a requirement.

### Communication Barriers

Leaders and providers described how negative attitudes and beliefs about research that are grounded in experiences of unethical research and inadequate health care could predispose patients to be mistrustful of researchers and fearful of precision medicine, whether in a research or clinical context. Providers thought that these attitudes and beliefs had the potential to distort how patients interpret information they receive about precision medicine and suggested developing messaging that led with and emphasized the benefits of precision medicine for ANAI people. Some participants thought that these conceptions would be less prevalent among younger patients who lacked direct experience with unethical research practices.

Provider: “… people really feel suspicious of Indian Health Services doing studies on ‘em. I have met people who have felt like things have been done to them in the name of research… I would say that 40 and over might be more suspicious… but I think the younger population would feel maybe more open to it, just because they’re probably—what they’ve seen of health care provided to them is a lot different than the way it’s been provided in the past. So there might be less—there might be more trust there.”

As described above, care in the selection of terms used to describe precision medicine is needed to avoid confusion and mistrust. In a related concern, some participants observed that older patients and those living in remote areas may have limited fluency in English; these individuals proposed that materials for communicating about precision medicine should be available in all languages spoken by the customer-owner population.

Patient: “If you want to get the word out on [precision medicine], you should have different types of interpreters of each different region, you know, and they speak it so that the Elders and the other people that don’t know English very well—I know most of them do, but there’s some that mostly speak Native tongue than English. So, to get the word out it’s—translate.”

Participants observed that precision medicine concerns inherently complex concepts and can involve research activities, such as extensive sharing of genomic data, that raise concerns for many ANAI communities. To be effective, communication efforts will need to account for these barriers to comprehension and address community sensitivities and concerns that could undermine participation in or use of precision medicine. Participants also speculated that these factors might interact with others, such as duration of clinical visits and accessibility of remote villages, to impede precision medicine communication efforts. For example, providers speculated that in order to communicate clearly about a topic as complicated as precision medicine might require face-to-face discussion with patients where questions, concerns, and other feedback could be received and responded to in real-time. However, clinical visits were thought to be too short and busy to incorporate meaningful discussion of precision medicine and its application in clinical or research settings. These challenges would be exacerbated for remote communities where the ratio of providers to patients is lower and alternatives to in-person discussion are expensive, unreliable, and/or impede or prevent bidirectional exchange.

Provider: “If we were going to institute something like this… then that would be a lot of work, a lot of time trying to explain that to people. Then there would be very little time left for the rest of the things we would have to do. Our visits with people in the exam rooms are packed, so we have to figure out a way to get the information to people that’s on their time, they can digest slowly, and think about it, and do more research.”

### Feedback

Members of all participant groups stated that SCF’s culturally-adapted, patient-centered approach to care and its emphasis on healthy patient-provider relationships encourage bilateral communication between providers and patients. Specifically, participants asserted that this model of care promoted egalitarian relationships and open discussion between patients and providers. This was contrasted with older models of care in which provider-patient relationships were hierarchical and feedback from patients was atypical. Patients also reported that durable relationships with providers help to build trust in the health care system and encouraged patients to recognize providers as sources of reliable information with whom they could share concerns and questions. Providers corroborated these statements, observing that they encourage and had come to expect discussion and questions from patients.

Leader: “[Providers] would still ask the same question: ‘What do our customer-owners [patients] think?’. Because of the SCF Nuka model of care, they’re already in relationship and [providers] would be thinking of their customer-owners, saying, ‘Hmm, if I said, ‘Okay, we’re doing precision medicine here,’ the customer-owner would say, ‘Well, what is that?’ and then be curious about that.”

Participants described many forms of feedback occurring within and between groups of patients and providers. For example, patients described how information about precision medicine that is displayed on television monitors located in clinic waiting areas might prompt patients to ask each other or their providers questions about precision medicine. In a similar way, brochures and posters were seen as catalysts for initiating conversations among patients and between patients and providers about precision medicine. The need for feedback among providers about precision medicine was also recognized. One leader proposed establishing regular debriefing opportunities to allow providers to discuss best practices and concerns related to clinical and research applications of precision medicine.

The focus groups and learning circles regularly hosted by SCF were viewed as particularly fruitful opportunities for bidirectional exchange about health-related issues. One patient noted that the unique benefit of these group discussions was that they allowed individual patients to hear and respond to a wider range of questions than they would have been able to ask during a one-on-one conversation with a provider. Providers also suggested that group discussions with patients could serve as platforms for sharing information and answering questions about precision medicine. Providers saw such discussions as starting points for more in-depth conversations with individual patients.

Patient: “When you [learn about precision medicine] in group settings, what’s nice is that we all think differently. When you get a group together and you collaborate, you get different insights and views in small little glimpses that you would’ve never thought about your own because we all think differently.”

Finally, participants observed that SCF’s commitment to bidirectional communication was evident in its tradition of seeking patient perspectives on organizational priorities. Providers suggested that community advisory councils, “listening posts” for capturing patient feedback, the organizational website and social media accounts, and other resources developed to enable patients to share their perspectives with organizational leadership could be repurposed as tools for promoting bidirectional exchange of information about precision medicine. Participants also proposed investing in additional resources to promote feedback, including a medical geneticist or other health care professional with relevant training who could participate in patient-provider discussions, answering questions and providing information and recommendations about precision medicine as needed.

## DISCUSSION

**FIGURE 2 |:**
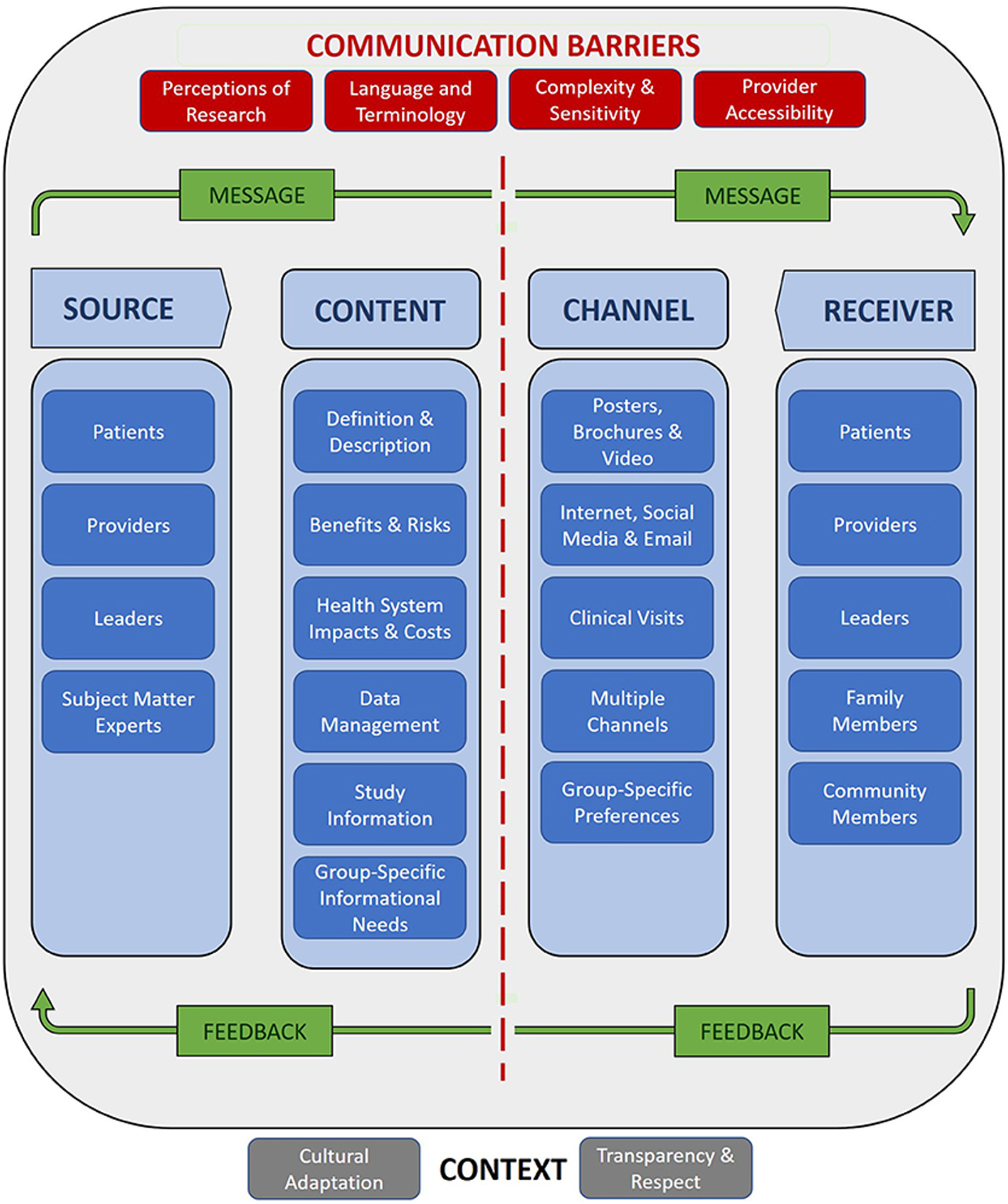
Southcentral Foundation precision medicine communication model.

This study explored the perspectives of SCF patients, providers, and leaders on communicating about the research and clinical applications of precision medicine. Participants identified potential sources and recipients of information; cataloged the essential information to be included in messaging about precision medicine; recommended communication channels based on the needs and preferences of specific groups; described how to account for cultural and institutional context through an adaptive and principle-based approach to communication; proposed strategies for overcoming barriers to effective communication; and, listed factors influencing and preferred means of engaging in bilateral exchange. These themes correspond to the components of the basic model of health communication depicted in Figure 1 above. Figure 2 populates the model components with participant observations and recommendations reported in the results section, providing a roadmap for communicating to SCF stakeholders about precision medicine.

Although patients, providers, and leaders each articulated a distinct set of informational needs, some of these needs were common to one or more groups. This intergroup agreement suggests an approach to developing a precision medicine communication plan that seeks to foreground content, prioritize channels, and adopt strategies that were deemed requisite or desirable by all stakeholder groups.

First, participants were unequivocal that accurate and complete information about precision medicine was needed in order to make informed decisions about whether to implement or engage in a precision medicine program. To meet this expectation, approaches to communicating about precision medicine will need to be in constant evolution as advances in research and clinical care alter data management practices (Prosperi et al., 2018; Hulsen et al., 2019), health system costs and impacts (Gavan et al., 2018; Ginsburg and Phillips, 2018), and the balance of perceived benefits and risks associated with precision medicine (Beskow et al., 2018; Lee et al., 2019).

Providing a clear definition and description of precision medicine—in general and as enacted through specific programs—will require, at minimum, clarification of core genetic and scientific concepts and distinguishing between precision medicine’s research and clinical applications. Results from this study suggest that participants often misunderstood these concepts and did not make this distinction. Similar findings have been reported in research with other populations (Catz et al., 2005; Christensen et al., 2010; Krakow et al., 2017; Anderson et al., 2018; Board, 2018). Parsing out the research and clinical components of precision medicine is made challenging by its translational orientation, which closes the distance between research and clinical care (Henderson et al., 2012; Wolf et al., 2018). This close relationship has advantages for patient care but can also lead to misconceptions—therapeutic and otherwise—where the benefits and risks associated with research are conflated with those unique to clinical care (Halverson and Ross, 2012; Perry et al., 2017). Health systems considering implementation of precision medicine programs will need to design communication plans that effectively differentiate between these components of precision medicine. This may be particularly challenging for learning health systems—where the boundary between research and clinical practice is porous by design—and for any health system that implements a precision medicine program with both clinical and research components.

Second, participants emphasized that efforts to communicate about precision medicine should disseminate information through multiple channels and account for age-specific channel preferences. Participants also demonstrated a general preference for in-person methods of communication, including face-to-face discussions during patient-provider encounters and learning groups. This disposition toward direct, interpersonal communication may derive from positive experiences with Southcentral Foundation’s relationship-based approach to care and the important role that narrative and interpersonal communication play in many ANAI cultures (Dillard et al., 2018). A substantial literature describes similar communication preferences across several ANAI communities and the effective use of narrative and in-person communication methods in health promotion and education efforts tailored for ANAI populations (Hodge et al., 2002; Vogel et al., 2013; Cueva et al., 2015, 2016). Strategies for communicating about precision medicine in this population should employ a multichannel approach to disseminating information while focusing resources on direct methods of communication (Hiratsuka et al., 2018b; Dirks et al., 2019).

Finally, members of all participant groups remarked on communication barriers and contextual factors—including negative perceptions of research, the geographic distribution and remote character of the patient population, and culturally-informed perceptions of clinical terminology—that would need to be accounted for when developing an approach to communicating about precision medicine at SCF. Many of these barriers and factors were also identified in prior research involving this and other ANAI populations (Pacheco et al., 2013; Beans et al., 2018; Dillard et al., 2018). There was a particular focus on the need to culturally-adapt the language and terminology used to describe precision medicine and to frame the goals and risks and benefits of precision medicine in ways that speak to community concerns about the conduct of research and clinical care.

SCF has extensive experience in tailoring clinical and research processes, and the language used to describe them, to the needs and preferences of its patient population (Driscoll et al., 2013; Starks et al., 2015; Hiratsuka et al., 2017a,b, 2018c, 2019a,b; Muller et al., 2017; Dillard et al., 2018). For example, considerable organizational resources are devoted to training clinicians and staffin the use of a “shared language” that encourages rapport among employees and between providers and patients and was referred to by participants (Eby, 2007; Gottlieb, 2013). Interpersonal communication at SCF is also guided by a set of communication standards that are used by clinicians and staffas a guide for interacting with patients. These standards echo the principle-based approach to communicating about precision medicine described by participants, and suggests that patients, providers, and leaders have internalized these concepts and come to expect their application in patient engagement. In addition, major research streams at SCF concern the exploration of community preferences regarding dissemination of research results and involve tailoring interventions developed for use in other populations to meet the needs, expectations, and preferences of patients (Starks et al., 2015; Hiratsuka et al., 2017a, 2018a,b,c, 2019b; Muller et al., 2017; Beans et al., 2018; Shane et al., 2018). Any effort to communicate about precision medicine at SCF will benefit from this organizational commitment to and competency in tailoring clinical and research practices to patient needs.

This study has strengths and limitations. By describing patient, provider, and leader perspectives on communicating about precision medicine in the context of a Tribal health care system utilizing a culturally-adapted, patient-centered medical home, these results represent a unique contribution to the empirical health communication literature. These findings also provide valuable insights that can be generalized for application in other health care systems—Tribal or otherwise—that employ patient-centered health care models and are considering implementation of precision medicine programs. Limitations include the use of convenience sampling approach that recruited participants employed or receiving care at an urban health care facility and an interview guide developed without explicit reference to health communication models. As a result of the sampling approach, findings may not represent the perspectives of patients, providers, and leaders living in rural areas. However, recruiting exclusively from an urban health care center may promote the generalizability of our results, since over 70% of ANAI people in the United States reside in urban areas (U.S. Census Bureau, 2010). Although the interview guide posed broad questions related to health communication it included no mention of the model or model components used to organize the study results. An instrument constructed around this model would likely have produced a dataset better targeted to the research questions. Model components and participant responses nevertheless coincided to a remarkable degree, suggesting close alignment between the core elements of models used by communication theorists and public conceptions of communication.

## CONCLUSION

The objective of precision medicine is to deliver the right treatment, in the right manner, to the right patient, at the right time. Much scholarship and research funding has been devoted to understanding how this objective might be achieved through approaches to research and clinical care that account for the role of genes, environment, and lifestyle in individual-level health, susceptibility to disease, and response to treatment. Far less attention has been paid to how key aspects of precision medicine might be communicated to patients, participants, providers, leaders, and other stakeholders within health care systems. As a result, little is known about the informational needs and communication preferences of organizations and individuals considering implementation of or participation in precision medicine programs.

The present study addresses this gap as it pertains to the patients, providers, and leaders of a specific Tribal health organization. Findings also suggest that communicating completely, accurately, and cogently about precision medicine will require an approach to communication that is as precisely tuned to individual needs as the research and medicine they describe. This *precision communication* approach recognizes that messaging about precision medicine is effective to the extent that it accounts for individual and organizational preferences regarding communication sources, recipients, content, and channels. As major precision medicine efforts continue to move forward, further health communication and implementation science research will be needed to develop the communication approaches needed to realize the full potential of these significant investments in research and medical care.

## Supplementary Material

Supplementary Materials
