# Supplementary material for: Community Perspectives on Communicating About Precision Medicine in an Alaska Native Tribal Health Care System
Source: Front Commun (Lausanne). Author manuscript; Available in PMC 2021 Jan 27. (PMC7839995; doi:10.3389/fcomm.2020.00070)
Supplement: Supplementary Materials [file NIHMS1652912-supplement-Supplementary_Materials.pdf]

Thank you for talking with me today. This interview will cover a few topics: your views on genetic medicine, precision medicine, how you see precision medicine fitting in at SCF, and how Alaska Native people should be approached about precision medicine. Your answers will be anonymous. The information that you share will help us understand how SCF might consider work in precision medicine in the future. We appreciate you taking the time to share with us.

1. What do you think about when you hear the terms 'genetics', 'genetic testing' or 'genetic medicine'?

2. Tell me about any experience you or your family has had with genetic tests?
   Probes:
   - Genetic testing is a part of routine prenatal care for pregnant mothers, do you have any type of concerns with type of testing?
3. Would you be comfortable talking with your provider about genetic tests?

Precision medicine is a new approach to disease prevention and treatment based on people's individual differences in environment, genes, and lifestyle.

Some examples of how Precision Medicine can be applied in clinic include: blood tests that can be used to assess if a person has an increased or decreased risk of developing common diseases, targeted cancer treatment, using a blood test to identify whether a specific medicine would work for an individual or a blood test that would identify what foods an individual's body can break down easier than others.

4. What are your thoughts on the use of Precision Medicine for the SCF health system?
   Probes:
   - How does Precision Medicine fit at SCF?
   - What are the benefits of Precision Medicine use for SCF?

5. What concerns would you have with Precision Medicine supporting your healthcare?

6. Would you be comfortable talking with your provider about Precision Medicine?

Note: Provide the NIH Precision Medicine Initiative Cohort Program show card

The NIH Precision Medicine Initiative Cohort Program goal is to engage a group of 1 million or more US research participants who will have information in their medical record related to biological samples, genetic data, and diet/lifestyle information available for their healthcare provider.

7. Do you think it is important for AI/AN to be included in this type of research? Why or why not?
   Probes:
   - What type of information should AI/AN people be given when being asked to be participate in this type of cohort?
   - What are your concerns about including and excluding AI/AN participants in the cohort?
   - How do you feel about the sharing of medical record related to biological samples? Genetic data? Diet/lifestyle information? These types of information being shared with a healthcare provider? These types of information being shared between healthcare providers?

8. Thinking about primary care at SCF and specialty care through the Alaska tribal health system, how should AI/AN people be approached about Precision Medicine?
   Probes:
   - What do leaders/providers/clinical staff/AIAN people need to know?

- What type of resources would you want to help you make decisions about including precision medicine in your healthcare at SCF?
- What materials would need to be developed by SCF?
- Who should share the information?
